# Wnt Drug Discovery: Weaving Through the Screens, Patents and Clinical Trials

**DOI:** 10.3390/cancers8090082

**Published:** 2016-09-01

**Authors:** Benjamin Lu, Brooke A. Green, Jacqueline M. Farr, Flávia C.M. Lopes, Terence J. Van Raay

**Affiliations:** Department of Molecular and Cellular Biology, University of Guelph, Guelph, ON N1G 2W1, Canada; boyang@mail.uoguelph.ca (B.L.); bgreen04@mail.uoguelph.ca (B.A.G.); jfarr01@mail.uoguelph.ca (J.M.F.); fcmlopes@yahoo.com.br (F.C.M.L.)

**Keywords:** Wnt, β-catenin, TOPflash, SUPERTOPFlash, HEK293, screen, inhibitors, patents, cancer, clinical trials

## Abstract

The Wnt signaling pathway is intricately involved in many aspects of development and is the root cause of an increasing number of diseases. For example, colorectal cancer is the second leading cause of death in the industrialized world and aberration of Wnt signaling within the colonic stem cell is the cause of more than 90% of these cancers. Despite our advances in successfully targeting other pathways, such as Human Epidermal Growth Factor Receptor 2 (HER2), there are no clinically relevant therapies available for Wnt-related diseases. Here, we investigated where research activities are focused with respect to Wnt signaling modulators by searching the United States Patent and Trade Office (USPTO) for patents and patent applications related to Wnt modulators and compared this to clinical trials focusing on Wnt modulation. We found that while the transition of intellectual property surrounding the Wnt ligand-receptor interface to clinical trials is robust, this is not true for specific inhibitors of β-catenin, which is constitutively active in many cancers. Considering the ubiquitous use of the synthetic T-cell Factor/Lymphoid Enhancer Factor (TCF/Lef) reporter system and its success in identifying novel modulators in vitro, we speculate that this model of drug discovery does not capture the complexity of in vivo Wnt signaling that may be required if we are to successfully target the Wnt pathway in the clinic. Notwithstanding, increasingly more complex models are being developed, which may not be high throughput, but more pragmatic in our pursuit to control Wnt signaling.

## 1. Introduction

The Wnt signaling pathway is one of the oldest signaling pathways in multicellular eukaryotes and is involved in many aspects of development and in the maintenance of stem cells [1,2,3]. Deregulation of this pathway is at the root of many diseases ranging from hair loss to osteoporosis to cancer and nervous system disorders [4,5,6]. Indeed, a cursory search of the literature on Wnt signaling will invariably turn up the phrase “Thus, the Wnt pathway is a potential therapeutic target for [insert favorite disease here]”. Moreover, given the ubiquity of this pathway in development and disease in combination with 19 Wnt ligands, 10 Frizzled receptors and a host of other co-receptors and extracellular modifiers [7], one would think that there would be several opportunities to specifically target a Wnt-related disease based on the combination of ligand and receptors. Towards this end, there are many high throughput screens that have identified some promising candidates [8,9,10,11,12], but there are currently no specific Wnt targeted therapies. Unfortunately, the results of many of these screens are not published prior to securing intellectual property rights. This makes it rather difficult to assess what potential therapies may be in the pipeline. Therefore, we attempted to obtain a view of where researchers are focusing their efforts in the battle against Wnt related disease. As aberrant Wnt signaling is involved in many kinds of diseases, we started by searching the United States Patent and Trade Office (USPTO) Patent and Patent Application databases (P/PA). This is by no means a comprehensive search for all molecules that modulate Wnt signaling. Instead, it provides a representation of where researchers are focusing their efforts. In addition, we also sought to understand what methods researchers are using to identify Wnt inhibitors and the status of Wnt modulators in the clinic.

## 2. Methods

### 2.1. USPTO

We focused on the USPTO Patents and Patent Application (P/PA) databases searching abstracts for “Wnt AND cancer” or “-catenin AND cancer” and combined these results with the general search term “Wnt inhibitor”. This produced a combined total of 674 P/PA (Table 1). These were then sorted to remove redundancies (e.g., a Patent Application that has become a Patent and multiple applications with the same title, where only the most recent application was kept. P/PA having no obvious reference to Wnt signaling modulation were removed (many applications make reference to the effect of a molecule on many signaling pathways in the lengthy descriptions of the invention). This filtering resulted in 165 Patents and 181 Patent Applications. Numerous molecules have several Patents and/or Patent applications assigned to the same company or individual and we therefore sought to combine these to the best of our ability to prevent over representation of some molecules. Finally, P/PA were removed that focused on the following: methods for genetic testing; secondary or general references to Wnt (e.g., the need to inhibit Wnt signaling to induce cardiomyocyte differentiation); inhibition or stimulation of another pathway that alters Wnt signaling; models of cancer; unknown mechanisms of action; multiple targets; miRNAs that affected multiple targets; biomarkers and finally single P/PA that incorporate several modulators, each with known targets (e.g., Patent 9045416 “WNT protein signalling inhibitors”).

### 2.2. ClinicalTrials.gov

Search for “Wnt” or “Catenin” or “DKK” resulted in 84 search results. Studies that incorporated non-specific inhibitors (e.g., NSAIDs) or looking for biomarkers were removed, resulting in 34 Clinical Trials referencing the inhibition or activation of Wnt signaling.

### 2.3. Wnt Screens

We focused primarily on screens that have been completed since 2005, searching the PubMed database for “Wnt[Title/Abstract] AND Inhibitor*[Title/Abstract] AND screen*[Title/Abstract] AND ‘2005’[Date-Publication]: ‘3000’[Date-Publication]” which returned 242 articles as of 22 June 2016. We scanned the abstracts of these 242 articles to identify papers that specifically screened for inhibitors of Wnt signaling, removing screens that identified inhibitors of Wnt signaling via other routes (e.g., screens for molecules that induced cardiomyocyte differentiation) among others.

## 3. Results

### 3.1. USPTO

The objective of the search was to obtain a broad overview on where researchers were focusing their efforts. While we attempted to be as comprehensive as possible, our search criteria had some limitations. First, our search and filtering criteria missed several well-known inhibitors. For example, a search of the P/PA databases for LGK974, a potent inhibitor of the Wnt secretion protein Porcupine [13] identified by researchers at Novartis only turned up in PA by Novartis (PA# 20150125857 “Cancer Patient Selection for administration of Wnt Signaling inhibitors using RNF43 status”). This application was subsequently removed from our list because of its focus on biomarkers to identify patients. LGK974 is currently undergoing Phase 1 clinical trials sponsored by Novartis. In another example, the general search term “Wnt inhibitor” failed to pick up several P/PA such as Patent# 9,096,587 “Triazole derivatives as Wnt signaling pathway inhibitors”. Unfortunately, a search for “Wnt AND Inhibitor” is too general, turning up 2768 hits. Adding the search term “AND Triazole” to this general string did pick up this patent, along with 242 others. Therefore, while acknowledging these limitations, we do believe our results provide a representation of the state of the field in identifying Wnt signaling modifiers.

#### 3.1.1. Overall

**Figure 1:**
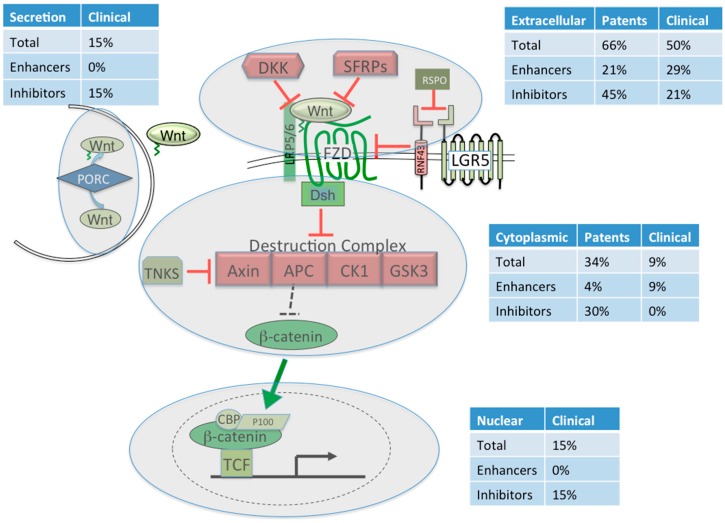
Patents and clinical trials involving the Wnt signaling pathway. Only the components of the Wnt pathway relevant to the patent or clinical trial searches are shown. Patents refer to novel compounds, peptides or proteins that modulate some aspect of the Wnt pathway. Red and green hued Wnt signaling components refer to the endogenous function of the protein as an inhibitor or activator of Wnt signaling, respectively. Grey transparent circles identify the space related to the percentage of patents or clinical trials occupying that space: Extracellular domain, cytoplasmic domain, nuclear domain or Wnt secretion domain. Percentages are relative to the total number of P/PA identified (103) or the number of clinical trials (34). See Table 2 for details on the enhancers and inhibitors in the different compartments. For the clinical trials, there are two unspecified targets and two that are targeting the epigenome, which together account for 11% of all clinical trials. Note that an absence of patent information for the Secretion and Nuclear domains does not mean there are no patents in these domains, just that they were not recovered from this search.

After filtering we ended up with 151 P/PA that were ultimately combined into 103 unique P/PA with high confidence as Wnt signaling modulators (Table 2; Table S1). The majority (66%) of all these modulators target the extracellular space, with DKK (25/103; 24%) being the most common subject for both inhibiting and enhancing the Wnt signaling pathway (Table 2, Figure 1).

The Wnt ligands were the next most popular extracellular subject (19%) followed by the Frizzled and LRP5/6 receptors (13% and 7%, respectively). The intracellular protein β-catenin tied with DKK as a popular choice for modulating the Wnt pathway, accounting for 24% of the P/PA (Table 2).

#### 3.1.2. Enhancers

Perhaps one of the most surprising finds was the effort put towards identifying enhancers, which was not part of our original search efforts. A subsequent search for Wnt enhancers or activators turned up a few more patents and patent applications (not shown), but the subjects of these patents were already covered under our original search and therefore do not offer any further information on the research space. Of the 103 P/PA, 74% were inhibitors of Wnt signaling, while 26% were Wnt signaling enhancers (27/103) (Table 2). Twenty-two of the 27 enhancers were concentrated around the ligand-receptor interface and of these 22, 12 were inhibitors of DKK. Inhibitors of DKK included antibodies (7/12); interfering peptides (2/12); dsRNA, shRNA, siRNA (2/12); and a small molecule inhibitor. The remaining 11 enhancers concentrated around the extracellular space including Wnt proteins (6/23); a Wnt-Frizzled chimera; overexpression of RSPO; inhibitors of TIKI1/2 and inhibitors of SOST.

The intracellular Wnt agonists concentrated around β-catenin stability by either inhibiting GSK3 (2/5); affecting the β-catenin-E-cadherin interaction; inhibition of its phosphorylation; or overexpression β-catenin itself. The vast majority of agonists appeared to be targeting bone growth, such as possible treatments for osteoporosis, although a couple of these P/PA targeted hair loss.

#### 3.1.3. Inhibitors

The ligand-receptor interface was also very popular for antagonizing Wnt signaling, most likely because of its accessibility by biomolecules. Of the 76 inhibitors identified in our search, 45 (59%) were targeted to inhibiting activation of the receptor. Methods used to antagonize receptor activation were fairly equally divided among antibodies to Wnt, Frizzled or LRP6; or the overexpression of soluble frizzled fragments or Wnt ligand peptides (Table 2; Table S1). While there is substantial diversity in the extracellular space with regards to inhibitors, this did not compare to β-catenin, which was the overall dominant candidate for inhibiting Wnt signaling accounting for 28% (21/76) of the P/PA focused on inhibiting Wnt signaling (Table 2). Many of these targeted the β-catenin-TCF-CBP interaction and the P/PA were equally represented by peptides that interfere with protein-protein interactions or small molecule inhibitors. We found six P/PA that targeted Axin, one that targeted Dishevelled and two that targeted Casein Kinase 1.

Some other interesting findings included the ratio of P/PA focused on chemicals compared to ones focusing on biomolecules (Table 3). Based on the relatively high number of high-throughput screens using small molecule libraries, we were expecting more of these types of compounds compared to biomolecules. However, biomolecules such as antibodies, peptides, polypeptides and full-length proteins dominated the intellectual property landscape accounting for 76% of the P/PA. Chemicals accounted for 20% and RNA mediated interference accounted for 4% of the P/PA. Lastly, much of the intellectual property is assigned to universities, hospitals and research institutes (48%), while private industry has 40% of the intellectual property, with the remaining 12% being unassigned in the Patent Applications (Table S1).

### 3.2. Clinical Trials

We identified 34 clinical trials registered with ClinicalTrials.gov that involve treatments to either inhibit or activate Wnt signaling (Table S2). Of these, 18 are/were in Phase 1/1b; five in Phase 1/2; eight in Phase 2; one in Phase 2/3; and two are unknown or inapplicable. Consistent with our findings in the P/PA search, one half of the clinical trials are using compounds that target the ligand-receptor interface (17/34; Table 4 and Table 5); however, the majority of these trials are trying to activate Wnt signaling via DKK1 neutralizing antibodies (9/17; 53%; Table 6). Of the nine trials using Dkk1 neutralizing antibodies to activate Wnt signaling, six are treating Multiple Myeloma related cancers: three of the trials are by Leap Therapeutics using a proprietary DKK1 neutralizing antibody DKN-01; and three by Novartis Pharmaceuticals using the well-characterized DKK1 neutralizing antibody BHQ880. Leap Therapeutics is also using their DKK1 neutralizing antibody to treat several other cancers (Table S2), but the functional role of DKK1 in these cancers is much less clear. In several cases, the same clinical trial is targeting multiple conditions such as Multiple Myeloma and Bone Disease (Table S2; Table 6). The majority of clinical trials using anti-DKK1 therapy are complete, so we should soon know the future of this field as a possible therapy.

Of the 19 trials focusing on inhibition of the Wnt pathway, none are using DKK proteins (Table 4), even though these were highly represented in the P/PA (Table 2). Five trials (15%) are using inhibitors of Porcupine, three of which are using the LGK/Wnt-974 small molecule [13]; four are inhibiting Frizzled receptors using a variety of tools including one neutralizing antibody; a Wnt5a mimic (2); and the anti-parasitic compound Niclosamide. Five clinical trials focus on inhibiting the CBP/β-catenin complex, with four of these using PRI-724, which specifically inhibits the interaction between β-catenin and cAMP response element-binding protein (CBP), but not the closely related homolog p300 [12,14]. The other β-catenin inhibitor is CWP232291, a non-specific small molecule that was discovered by Theriac Pharmaceutical Corporation. Finally, the types of conditions being treated are wide spread, but mainly involve cancers, with colorectal carcinoma being treated in seven of the 19 trials (37%; Table 6 and Table 7).

### 3.3. Wnt Screens

We identified 76 screens that were specifically searching for modifiers of Wnt signaling (Table 8). These were broken down into in vitro cell based screens (N = 56), in vitro substrate based (N = 5), in silico based (N = 9) and model based (N = 6). Of the cell based in vitro screens, 52 used the TCF/Lef reporter based systems. Of these 52, 31 were HEK293 cells, seven were the colorectal cancer cell lines HCT116 (5) or SW480 cells (2), two were the mouse testis cell line TM3 and 12 were singular cell lines (Table 8). While the size of the library that was used for screening was not always disclosed and different groups may have used the same library, we estimate that nearly three million molecules have been screened using the TCF/Lef reporter based system. The remainder of the screens were varied in their models and approach, encompassing virtual screen techniques to identify potential modifiers of the DKK1 interactions to the use of reporters in zebrafish scales [15,16]. In one case, the use of phage display allowed for the screening of 12 billion bicyclic peptides that might interact with the β-catenin ARM repeats [17].

## 4. Discussion

The involvement of Wnt signaling in so many biological processes and diseases makes this pathway a difficult yet obvious target for therapeutic intervention and there are numerous reviews on this subject [5,88,89,90,91,92,93,94,95,96]. Thus, any successful therapy will ultimately lead to significant financial gains. Inevitably this results in the protection of intellectual property and so the Patent and Patent Application databases are excellent resources to identify where researchers are focusing their efforts with respect to Wnt signaling inhibitors and enhancers. However, the legal language used in patent law is unlike the scientific language, making it somewhat difficult for the average scientist to understand exactly what is being protected in a Patent. At the risk of exposing our ignorance in the field of patent law, we attempted to find patents and patent applications to better understand the current status of Wnt signaling therapeutics.

Our first observation is that most of the intellectual property is focused around the ligand-receptor interaction. This was somewhat unexpected considering that mutations in the APC and β-catenin genes account for greater than 90% of all colorectal cancers, which is the second leading cause of death in the developed world [97]. The reason for this is likely two-fold. First, the clinical efficacy of inhibiting a ligand-receptor interaction with antibodies has been clearly demonstrated in the Receptor Tyrosine Kinase (RTK) pathway field with clinically validated antibodies inhibiting HER2, EGFR, VEGFR, PDGFR and FGFR signaling in numerous cancers [94,98]. Second, there is evidence that inhibition of the extracellular Wnt ligand-receptor interaction is effective in reducing tumorigenicity in cancers that harbor APC mutations [99]. Moreover, there are numerous cancers with downregulation of extracellular Wnt inhibitors such as DKK1, sFRP and Wif1 that make the development of therapies targeting the Wnt ligand-receptor complex low hanging fruit [100,101,102]. Based on the number of clinical trials, this appears to be a viable approach for several Wnt-related diseases, but its efficacy in treating cancers with downstream activating mutations will need to be determined.

Perhaps the most interesting finding was that inhibition of Wnt signaling via β-catenin was the focus of the majority of P/PA (22/103), but there are only five clinical trials in this area, one of which is a non-specific inhibitor and four using the same compound, PRI-724, to inhibit the β-catenin-CBP interaction [12,14]. The disparity between P/PA and clinical investigations with respect to β-catenin is most likely due to the central role this protein plays in the Wnt signaling pathway. Systemic inhibition of β-catenin would likely have significant, non-specific effects across a range of tissues. This is why PRI-724 may be clinically successful, as it specifically inhibits the interaction between β-catenin and CBP, which would target the colorectal cancer stem cell but not healthy stem cells [14].

β-catenin transcriptional activity is central to the majority of the assays used to identify compounds that alter Wnt signaling (Table 8). The use of an artificial reporter construct containing synthetic, multimerized TCF/Lef binding sites such as TOPFLASH and SuperTOPFLASH [103,104] has been nearly universally adopted as the way to identify inhibitors of Wnt signaling in vitro (Table 8). Essentially, stabilization of β-catenin will result in its interaction with TCF/Lef transcription factors, activating the reporter. For screening purposes, this easily becomes automated and economical, with the ability to rapidly screen thousands of compounds to ultimately focus on a few of the highest scoring compounds [22,52,58,59,67,69]. Indeed, the evidence from Table 8 is clear that this method is successful in identifying Wnt modulators, but unfortunately they have yet to transform into clinically viable therapies (with the exception of PRI-724).

We hypothesize that there are two distinct, yet overlapping mechanisms at play. The first is rather obvious, that a synthetic reporter cannot simply capture the complexity of the Wnt signaling pathway. The second mechanism revolves around the Goldilocks hypothesis and the “just-right” amount of Wnt signaling required to induce or maintain tumorigenesis [105,106,107]. The authors in these two independent studies found that there is a highly significant correlation between the level of Wnt signaling, the position of the tumor in the colon and the promotion of tumorigenesis. In the proximal colon, APC mutations that select for 2–3 β-catenin binding sites are required for tumorigenesis, while in the distal colon APC mutations select for 0–1 β-catenin binding domains [105,106,107]. The main point is that too much intracellular Wnt signaling may actually be detrimental for CRC promotion. In screening for inhibitors our natural tendency is to select for the most potent inhibitors [108], but according to these studies, this may not be the best approach.

Other screening approaches are being proposed to tackle the first problem of complexity. For example, antagonists of specific Wnt-FZD combinations might be more selective for certain cancers compared to general β-catenin antagonists. Indeed, the FZD receptor has many advantages that could be used for high-throughput in vitro screening [109]. Another cell-based approach involves biomarker screening, where the expression of selected genes is monitored to identify compounds of interest [110]. If the second mechanism of the “just-right” hypothesis holds true, then this will require more intricate models, where aberrant Wnt signaling needs to be reduced back to baseline levels and not necessarily eliminated. Such models will typically incorporate whole organisms, but the high throughput nature of these screens becomes problematic. Nonetheless, several whole organism models have been used to screen for Wnt inhibitors (Table 8) but it may be too early to tell if any of the compounds identified in these screens will make it to the clinic. An alternative to the whole animal is the use of organoids. In the case of prostrate cancer and CRC, organoids are being developed that recapitulate the normal prostrate or intestinal structure or the tumor [111,112]. These are also being grown in a microarray platform ultimately allowing for novel drug-discovery [113].

## 5. Conclusions

Our comparison of the patenting and clinical field with respect to modulators of Wnt signaling has yielded some insight. First, inhibiting or enhancing Wnt signaling at the ligand-receptor level appears to have more traction. Considering the strong potential for clinical efficacy, we predict that the number and combination of ligands and receptors available for intellectual property protection will make this an active and attractive area for further investigation and investment. Second, and perhaps more importantly, the intellectual protection of real estate covering inhibitors of β-catenin is extensive, but this has not proven to be clinically viable, yet. We argue that the current methods used to identify modulators of Wnt signaling for clinical use do not capture the complexity of Wnt signaling that is intricately involved in many biological processes. More effective second generation screening mechanisms are required to capture this complexity and indeed some are already in use [15,82,83,86]. Next generation screens include the use of organoids or whole animal models, which may not necessarily be high throughput but may be more efficacious and pragmatic in the long run [12,111,112,114,115].

## Figures and Tables

**Table 1:** General search results of the USPTO patent and patent application databases.

Search Term	“(ABST/cancer AND ABST/Wnt)” or “(ABST/cancer AND ABST/-catenin)”	Wnt Inhibitor
Patents	94	101
Patent Application	203	276
Total	297	377

**Table 2:** Breakdown of unique patents and patent applications post filtering.

Cellular Space	Subject of the Patent	Total	Effect on Wnt signaling
Extracellular	N = 68 (66%)		
	DKK as a target	12	Enhance
	DKK as a therapeutic	13	Inhibit
	Wnt ligand as a target	11	Inhibit
	Wnt ligand as a therapeutic	7	Enhance
	Wnt4a Patent #6165751	1	Unknown ^1^
	Frizzled/Soluble Frizzled	13	Inhibit
	LRP5/6	7	Inhibit
	RSPO as a target	1	Inhibit
	RSPO as a therapeutic	1	Enhance
	TIKI1/2 as a target	1	Enhance
	SOST as a target	1	Enhance
Intracellular	N = 35 (34%)		
	β-catenin as a target	21	Inhibit
	β-catenin as a therapeutic	2	Enhance
	β-catenin Patent PA #20050171005	1	Inhibit and Enhance ^2^
	Axin1/2 as a target	6	Inhibit
	Dishevelled as a target	1	Inhibit
	Casein Kinase 1 as a target	2	Inhibit
	GSK3 as a target	2	Enhance
Total		103	

**^1^** In Patent #6165751 “HLDAT86 polynucleotides”, the function of Wnt4a is unclear. **^2^** Patent Application #20050171005 “Methods and compositions for modulating β-catenin phosphorylation” identifies both enhancers and inhibitors of β-catenin.

**Table 3:** Types of compounds identified to modulate Wnt signaling.

Method	Number of Patents	Percentage of Patents
Antibodies	25	25%
RNA mediated	4	4%
Polypeptides/proteins	53	51%
Chemicals	21	20%
Total	103	100%

**Table 4:** Wnt signaling inhibition targets.

Target	Number of Clinical Trials	Clinical Trial from Table S2
Porcupine	5	1, 2, 3, 13, 15
Wnt Ligands	1	23
Frizzled Receptor	4	6, 9, 12, 19
LRP5/6	2	24, 25
CBP/β-catenin	5	4, 8, 10, 16, 18
Epigenetic	1	21
Unspecified	1	11
Total	19	

**Table 5:** Wnt signaling enhancer targets.

Target	Number of Clincal Trials	Clinical Trial from Table S2
Wnt Ligands	1	5
Dkk	9	22, 26, 27, 28, 29, 31, 32, 33, 34
GSK3	3	7, 14, 17
Epigenetic	1	30
Unspecified	1	20
Total	15	

Clinical Trial #20: Specific targets are not identified; Clinical Trial #21 targets are the promoters of several Wnt genes (Sfrp2, Sfrp5 and Wnt5a); Clinical Trial #30 is just Wnt target genes.

**Table 6:** Clinical trials involving enhancers of Wnt signaling.

Condition	Number of Clinical Trials	Clinical Trial from Table S2
Osteoporosis	2	5, 31
Osteopenia	1	31,
Alopecia	2	7, 20
Male Pattern Baldness	1	7,
Alzheimer’s disease	1	14
Leukemia	1	17
Multiple Myeloma	6	22, 27, 28, 29, 33, 34
Bone Disease *	1	27
Renal Insufficiency *	1	28
Cholangiocarcinoma	1	26
Gastro-esophageal Cancers	1	32
Gall Bladder Cancer	1	26
Bile Duct Cancer	1	26
Total	20	

* In combination with treatments for Multiple Myeloma.

**Table 7:** Clinical trials involving inhibition of Wnt signaling.

Condition	Number of Clinical Trails	Clinical Trial from Table S2
Colorectal Carcinoma	7	1, 3, 4, 6, 12, 21, 30
Squamous Cell Carcinoma	2	2, 32
Head and Neck	1	2,
Pancreatic Cancer	2	3, 8,
Breast Cancer	2	6, 9,
Myeloid Leukemia	2	10, 16
Basal Cell Carcinoma	1	11,
Synovial Sarcoma	1	19,
Non-Small Cell Lung Cancer	1	22
Solid Tumors	5	13, 15, 18, 22, 23
Prostate Cancer	4	6, 9, 24, 25
Total	28	

**Table 8:** Assays for discovering modulators of Wnt signaling.

Discovery Platform	Number of Assays	Assay	Reference
**Cell Based**			
HEK293	31	TCF/Lef reporter	[18,19,20,21,22,23,24,25,26,27,28,29,30,31,32,33,34,35,36,37,38,39,40,41,42,43,44,45,46,47,48]
HEK293	1	β-catenin-luciferase stability	[49]
HCT116	5	TCF/Lef reporter	[50,51,52,53,54]
SW480	2	TCF/Lef reporter	[55] USPTO Patent# 8987298 ^1^
TM3	2	TCF/Lef reporter	[13] USPTO PA# 20110237573 ^2^
Others ^3^	12	TCF/Lef reporter	[56,57,58,59,60,61,62,63,64,65,66,67]
U2OS	1	Nuclear β-catenin	[68]
U2OS	1	Frizzled-1 GFP endocytosis	[69]
Preosteoblasts	1	Nuclear β-catenin	[70]
**Substrate based**			
Elisa-based	3	Inhibitors of β-catenin-substrate interaction ^4^	[71,72,73]
Elisa-based	1	GST-GSK3 with ADP-Glo	[74]
Biotinylated β-cat ARM repeats	1	Phage Display	[17]
**In Silico Based**			
β-catenin-TCF 3D model	1	Structure based screen	[53]
β-catenin-TCF 3D model	1	NMR screening	[75]
Dvl PDZ 3D model	3	Structure based screen	[76,77,78]
DKK-LRP6 3D model	1	Structure based screen	[16]
FZD8-Wnt8 3D model	2	Structure based screen	[79,80]
Wnt3a 3D model	1	Structure based screen	[81]
**Model Based**			
Xenopus Egg Extract	1	Axin-renilla luciferase: β-catenin-Firefly luciferase ratio	[82]
Zebrafish	4	Phenotypic screen	[83,84,85,86]
Zebrafish Ex vivo scales	1	SP7: Luciferase	[15]
*C. elegans*	1	β-catenin dependent QL.D cell migration phenotype	[87]

^1^ Indazole inhibitors of the Wnt signal pathway and therapeutic uses thereof; ^2^ N-(hetero)aryl, 2-(hetero)aryl-substituted acetamides for use as Wnt signaling modulators; **^3^** A375, A549, CLL, H1703, DBTRG, DLD1, Drosophila C18, HeLa, HepG2, HT1080, HT22, HuH6, and iPSC-NPCs; ^4^ Substrates: BCL2, and TCF.

## Supplementary Materials

The following are available online at http://www.mdpi.com/2072-6694/8/9/82/s1, Table S1: USPTO patent and patent applications ligand and receptor interface P/PA, Table S2: Clincal Trials.
