# Supplementary material for: Wnt Drug Discovery: Weaving Through the Screens, Patents and Clinical Trials
Source: Cancers (Basel). 2016 Sep 1;8(9):82. doi: 10.3390/cancers8090082 (PMC5040984; doi:10.3390/cancers8090082)
Supplement: Supplementary file 1 [file cancers-08-00082-s001.pdf]

Supplementary Table 1: USPTO Patent and Patent Applications
Ligand and Receptor Interface P/PA

Compound/Category	Title or Example Thereof for Multiple Patents	Inhibitor or Enhancer	Molecular target	Assignee	Patents and/or Patent Applications
Dkk protein	Therapeutic implications of dickkopf affecting cancer stem cell fate	Inhibition	LRP5/6	Institute for Medical Biomathematics	8762069
Dkk protein	Use of WNT inhibitors to augment therapeutic index of chemotherapy	Inhibition	LRP5/6	The Board of Trustees of the Leland Stanford Junior University	7803783
Dkk protein	Human Zven1 DNA, Peptides and polypeptides and Antibodies that bind human Zven1	Inhibition	Wnt ligands	ZymoGenetics Inc	6485938, 6756479, 6828425, 7122349, 7179787, 7662641, 7691585, 7691610,
Dkk genes	Inhibitor protein of the wnt signal pathway	Inhibition	Wnt ligands/ LRP5/6 receptor	Deutsches Krebsforschungszentrum Stiftung des Offentlichen Rechts	6844422, 7138508, 8461309, 8536311
DKK inhibitor	Composition of tumor-associated peptides and related anti-cancer vaccine for the treatment of glioblastoma (GBM) and other cancers	Enhancer	Dkk1	Immatics Biotechnologies GmbH	8895514, 8653035, 8961985, 20150125478,
Dkk protein	Prokaryotic expression of soluble, active Dkk	Inhibition	LRP5/6	St. Jude Children's Hospital	8470554
Dkk protein	Human dickkopf-related protein and nucleic acid molecules and uses therefor	Inhibition	Wnt ligands/ LRP5/6 receptor	Millennium Pharmaceuticals	7057017, 7579168, 7645451, 8202966, 20100093978
Dkk1 protein	WNT FAMILY-DERIVED PEPTIDES AND USE THEREOF	Inhibition	LRP5/6	CAREGEN CO., LTD.	20140309157
Dkk1 protein	METHOD FOR PREPARING INDUCED PARAXIAL MESODERM PROGENITOR (IPAM) CELLS AND THEIR USE	Inhibition	LRP5/6	INSERM, ASSOCIATION FRANCAISE CONTRE LES MYOPATHIES, UNIVERSITE DE STRASBOURG, CNRS	20140363886
DKK1 protein	METHOD FOR FORMING ENDOTHELIAL CELLS	Inhibition	LRP5/6	Korea Advanced Institute of Science and Technology (KAIST)	20150368617
Dkk1 and Dkk3 dsRNA	Compositions and methods for inhibiting cell migration	Enhancer	Dkk1/3	The Burnham Institute	7833980, 8039255
Dkk1 Antagonist	Inhibitors of Dkk-1	Enhancer	Dkk1	Administrators of the Tulane Educational Fund	20080085281
DKK1 antibody	Use of Anti-DKK-1 Monoclonal Antibodies for the Treatment of Liver Cancer	Enhancer	Dkk-1		20120201810

Compound/Category	Title or Example Thereof for Multiple Patents	Inhibitor or Enhancer	Molecular target	Assignee	Patents and/or Patent Applications
DKK1 antibody	DKK1 ANTIBODIES AND METHODS OF USE	Enhancer	Dkk-1		20130209475
DKK1 antibody	Antibodies to Dkk-1	Enhancer	Dkk1	Amgen	7709611
DKK1 antibody	Treatment involving Dkk-1 or antagonists thereof	Enhancer	Dkk1	Genetech, Inc.	20030165501
DKK1 antibody	Antibodies that bind human Dickkopf-1 proteins	Enhancer	Dkk1	Millennium Pharmaceuticals	7446181, 20100093056
DKK1 antibody	Antibodies specific for Dkk-1	Enhancer	Dkk1	Merck Sharp & Dohme Corp.	7994293
DKK1 antibody and over-expression of Wnt-3a	Overexpression of Wnt ligands and treatment of lytic bone diseases	Enhancer	Dkk1/wnt3-a	The Board of Trustees of the University of Arkansas	8501702
Dkk1 fragment	Compositions and methods for inhibiting cell migration	Enhancer	Dkk1	The Burnham Institute	7465585
DKK1 protein	INHIBITION OF HAIR FOLLICLE GROWTH BY THE WNT INHIBITOR DKK1	Inhibition	LRP5/6		20120165270
Dkk1 protein	Differentiation of human embryonic stem cells	Inhibition	LRP5/6	LifeScan, Inc.	9096832
DKK1 protein	Molecules, Compositions and Methods to Treat Organ Scarring	Inhibition	LRP5/6		20150337021
DKK1 shRNA or siRNA	shRNA Materials and Methods of Using Same for Inhibition of DKK-1	Enhancer	Dkk1	The Regents of the University of Michigan	20080293053
Dkk1, Dkk2 protein	NOVEL METHODS TO REGENERATE HUMAN LIMBAL STEM CELLS	Inhibition	LRP5/6	THE REGENTS OF THE UNIVERSITY OF CALIFORNIA	20150175965
Frizzled 7 expression	Frizzled proteins and detection and treatment of cancer	Inhibition	Frizzled-7	Rhode Island Hospital	7867705

Compound/Category	Title or Example Thereof for Multiple Patents	Inhibitor or Enhancer	Molecular target	Assignee	Patents and/or Patent Applications
Frizzled antibody (OMP-18R5) and Soluble Frizzed8	Combination Therapy for Treatment of Cancer	Inhibition	Receptor		20150132301
Frizzled antibody or polypeptide or lipid or small molecule	Frizzled-binding agents and their use in screening for WNT inhibitors	Inhibition	Frizzled	OncoMed Pharmaceuticals Inc.	8551789, 7982013, 8507442, 9168300, 20140242078
Frizzled, Soluble	Wnt antagonists and their use in the diagnosis and treatment of Wnt-mediated disorders	Inhibition	Wnt	Genentech Inc.	7947277
Frizzled, Soluble	Use of FZC18-Containing Collagen 18 Polypeptides for the Treatment, Diagnosis and Outcome Prediction of Diseases	Inhibition	Wnt ligands	INSERM, Paris	20100316616
Frizzled, Soluble	Wnt antagonists and methods of treatment and screening	Inhibition	Wnt ligands and Frizzled receptors	OncoMed Pharmaceuticals Inc.	9157904
Frizzled, Soluble (FRP)	Human FRP and fragments thereof including methods for using them	Inhibition	Wnt ligands	USA dept Health and Human Services	7183377
Frizzled, Soluble (FRP)	Polynucleotides encoding human FRP and fragments thereof	Inhibition	Wnt ligands	USA dept Health and Human Services	6479255
Frizzled, Soluble (Frzb)	Method of modulating tissue growth using Frzb Protein	Inhibition	Wnt ligands	USA dept Health and Human Services	6924367, 7049291, 7563596
Frizzled, Soluble (sFRP-1)	Differentiation of stem cells into dopaminergic cells	Inhibition	Frizzled	The United States of America, as represented by the Secretary, Department of Health and Human	8628962
Frizzled10 antibodies	Anti-FZD10 monoclonal antibodies and methods for their use	Inhibition	Frizzled-10	Omeros Corporation	9102724
Frizzled3/8/9 antibodies and sFRP1/2/3	METHODS AND COMPOSITIONS FOR NERVE REGENERATION	Inhibition	Frizzled-3/8/9	The University of Chicago	20080299135
Frizzled7 antibodies	Wnt proteins and detection and treatment of cancer	Inhibition	Frizzled-7	Rhode Island Hospital	8158761
LPR5/6 Receptor antagonist (Apcdd peptide)	Use of WNT inhibitor to inhibit angiogenesis in the CNS	Inhibition	LRP5 and Wnt3a		20110189097

**Compound/Category**	**Title or Example Thereof for Multiple Patents**	**Inhibitor or Enhancer**	**Molecular target**	**Assignee**	**Patents and/or Patent Applications**
LRP5/6 and Dkk proteins	Compounds and assays for controlling Wnt activity	Inhibition	LRP5/6 and Dkk	Enzo Biochem, Inc.	9052324
LRP5/6 antibody	Methods of using an antibody to inhibit WNT-mediated cardiac remodeling	Inhibition	LRP5/6	Vanderbilt University	8790650, 9175090, 9303087
LRP5/6 antibody	Antibodies That Inhibit WNT Signaling and Methods of Using The Same	Inhibition	LRP5/6		20120276089
LRP6 antibody	Anti-LRP6 antibodies	Inhibition	LRP6	Genentech, Inc.	8846041
LRP6 antibody	MOLECULES AND METHODS FOR MODULATING LOW-DENSITY-LIPOPROTEIN RECEPTOR-RELATED PROTEIN 6 (LRP6)	Inhibition	LRP6	Novartis	20100254980
LRP6 antibody	Monoclonal antibodies that inhibit the wnt signaling pathway and methods of production and use thereof	Inhibition	LRP6	The Board of Regents of the University of Oklahoma	20120045437
RSPO 1 protein	COMPOSITIONS AND METHODS RELATING TO INDUCTION OF INTESTINAL STEM CELL HOMEOGENESIS AND/OR REGENERATION	Enhancer	ligand-Receptor	The Regents of the University of Michigan	20160000865
RSPO Antibody	Anti-RSPO1 antibodies	Inhibition	RSPO1	Genentech, Inc.	8802097
SOST antibody	Antibodies that specifically bind SOST peptides	Enhancer	SOST	Stowers Institute for Medical Research	7893218, 7968301, 8168761, 8173125, 8546545, 9321835
Tiki 1/2 antibodies	Tiki1 and Tiki2, Wnt Inhibitors	Enhancer	Tiki1 and Tiki2	Children's Medical Center Corp.	20130101582
Wnt 7a/b proteins	WNT LIGANDS INVOLVED IN BLOOD-BRAIN BARRIER DEVELOPMENT AND USES THEREFOF	Enhancer	Frizzled	President and Fellows of Harvard College	20100199362
Wnt antibody	Wnt and frizzled receptors as targets for immunotherapy in head and neck squamous cell carcinomas	Inhibition	Wnt1	University of California Oakland	7682607
Wnt antibody	Wnt and frizzled receptors as targets for immunotherapy in head and neck squamous cell carcinomas	Inhibition	Wnt14	University of California Oakland	7713526

Compound/Category	Title or Example Thereof for Multiple Patents	Inhibitor or Enhancer	Molecular target	Assignee	Patents and/or Patent Applications
Wnt antibody	METHODS FOR TREATING CANCER BY INHIBITING WNT SIGNALING	Inhibition	Wnt1	Regents of the University of California, Oakland	20090304695
Wnt antibody	WNT-Binding Agents and Uses Thereof	Inhibition	Wnt1,2,2b,3,3a,7a,7b,8a,8b,10a and 10b	OncoMed Pharmaceuticals Inc.	20130045209
Wnt antibody	Mammalian Wnt polypeptide-5	inhibition	Wnt5	ZymoGenetics Inc	20030017980
Wnt inhibiton polypeptide	NON-ACTIVATED WNT INHIBITION POLYPEPTIDES AND METHOD FOR PREPARING THE SAME	Inhibition	Wnt ligand		20090325866
Wnt polypeptides (Mini-Wnt	WNT COMPOSITIONS AND METHODS OF USE THEREOF	Inhibition	Frizzled and LRP5/6	The Board of Trustees of the Leland Stanford Junior University	20140200179
Wnt protein	Use of Wnt Agents to Prevent Hypoxic Injury	Enhancer	Frizzled	The Board of Trustees of the Leland Stanford Junior University	20140141061
Wnt receptor decoy (sLRP6E1E2)	ANTI-CANCER COMPOSITIONS CONTAINING WNT DECOY RECEPTOR	Inhibition	Wnt ligand	INDUSTRY-UNIVERSITY COOPERATION FOUNDATION HANYANG UNIVERSITY	20140315825
Wnt-10 protein	WNT10-derived peptide and use thereof	Enhancer	Frizzled	Caregen Co., Ltd.	8497241
Wnt-5a peptide	Peptide ligand to impair cancer cell migration	Inhibition of Canonical	Receptor	Wntresearch AB	8674060
Wnt-Fzd chimera	Wnt-frizzled chimera	Enhancer	Pathway activation	Wyeth	20070072238
Wnt1/2/3a meroduplex RNA	NUCLEIC ACID COMPOUNDS FOR INHIBITING WNT GENE EXPRESSION AND USES THEREOF	Inhibition	Wnt1/2/3A	MDRNA, Inc	20100055784
Wnt3A protein	Use of liposomal Wnt composition to enhance osseointegration	Enhancer	Frizzled and LRP5/6	The Board of Trustees of the Leland Stanford Junior University	8809272
Wnt3A protein	Methods of promoting cardiac cell proliferation	Enhancer	Frizzled	Hydra	20050261189

Compound/Category	Title or Example Thereof for Multiple Patents	Inhibitor or Enhancer	Molecular target	Assignee	Patents and/or Patent Applications
Wnt4a protein	HLDAT86 polynucleotides	not specified	Frizzled receptors	SmithKline Beecham P.L.C.	6165751
Wnt7a polypeptide	WNT COMPOSITIONS AND THERAPEUTIC USES OF SUCH COMPOSITIONS	Enhancer	Frizzled and LRP5/6	Fate Therapeutics Inc	20150099708, 20140142046
Wnt7a protein	COMPOSITIONS, METHODS AND KITS FOR DETECTING AND TREATING CANCER	Inhibition	Frizzled	University of Miami	20110039788

Supplementary Table 1: USPTO Patent and Patent Applications
beta-catenin P/PA

Compound/Category	Title or Example Thereof for Multiple Patents	Inhibitor or Enhancer	Molecular target	Biomolecule or Chemical	Assignee	Patents and/or Patent Applications
bc19 like protein w/ mutations	.beta.-catenin nuclear localized protein	Inhibition	β-catenin	Biomolecule	Kyowa Hakko Kogyo Co	7358348
BCL9 HD2 helix	TARGETING DEREGULATED WNT SIGNALING IN CANCER USING STABILIZED ALPHA-HELICES OF BCL-9	Inhibition	β-catenin	Biomolecule	Dana-Farber Cancer Institute Inc.	20140113857
compound	Compounds useful for treatment of cancer, compositions containing the same, and methods of their use	Inhibition	β-catenin-TCF4 -CBP complex	Chemical	Choongwae Pharma Corp	6762185
debromohymenialdesine (dBHD) and dBHD analogs	Wnt signaling inhibitors, and methods for making and using them	Inhibition	β-catenin-TCF complex	Chemical	The Regents of the University of California	8304408
disulfonamide derivatives	Derivatives of fluorene, anthracene, xanthene, dibenzosuberone and acridine and uses thereof	Inhibition	β-catenin	Chemical	Dogwood Pharmaceuticals, Inc.	8129519, 9120754
Dvl, Wnt, phosphatase PP2A, CKI7, phosphatase inhibitors, FRAT1	Methods and compositions for modulating beta-catenin phosphorylation	Enhancer or Inhibition	β-catenin phosphorylation on Ser45	Biomolecule		20050171005
GSK3 inhibitor	ACTIVATED WNT-BETA-CATENIN SIGNALING IN MELANOMA	Enhancer	β-catenin	Chemical	Yale University & University of Washington	20110293750
LXXLL peptide	Compounds and methods for modulating .beta.-catenin mediated gene expression	Inhibition	β-catenin	Biomolecule	Adherex Technologies	6677116, 6303576
methods to modulate Fbp1 activity	NOVEL UBIQUITIN LIGASES AS THERAPEUTIC TARGETS	Inhibition	β-catenin	Biomolecule	New York University	20090104642, 20050079558
Nefopam	Method of treating scars and .beta.-catenin-mediated disorders using Nefopam compounds	Inhibition	β-catenin	Chemical	The Hospital for Sick Children	8957107
non-peptide reverse-turn mimics	Reverse-turn mimetics and method relating thereto	Inhibition	β-catenin-TCF4 -CBP complex	Chemical	Choongwae Pharma Corp.	8049008, 8729262, 8138337, 8318738, 7932384, 8101751, 8106049, 7232822, 7566711, 7576084, 7585862, 7671054
oligomers (w specific formulas)	Compounds for the modulation of beta-catenin expression	Inhibition	β-catenin mRNA	Biomolecule	Enzon Pharmaceuticals Inc. and Santaris Pharma	7915401, 8039446
Oxazole and thiazole small molecules	Oxazole and thiazole compounds as beta-catenin modulators and uses thereof	Inhibition	β-catenin	Chemical	New York University	9173871, 8252823
peptides that bind β-catenin such as cadherins	Methods and therapeutic compositions for treating cancer	inhibition	β-catenin	Biomolecule	2002	20020045591
peptidomimetic macrocycle (contains a helix)	PEPTIDOMIMETIC MACROCYCLES	Inhibition	β-catenin-TCF4 -CBP complex	Biomolecule		20130072439

Compound/Category	Title or Example Thereof for Multiple Patents	Inhibitor or Enhancer	Molecular target	Biomolecule or Chemical	Assignee	Patents and/or Patent Applications
quinazoline derivatives	AMINO-SUBSTITUTED QUINAZOLINE DERIVATIVES AS INHIBITORS OF BETA-CATENIN/TCF-4 PATHWAY AND CANCER TREATMENT AGENTS	Inhibition	β-catenin-TCF complex	Chemical	Wyeth	20090004185
RNAi agent	ORGANIC COMPOSITIONS TO TREAT BETA-CATENIN-RELATED DISEASES	Inhibition	β-catenin	Biomolecule	NOVARTIS AG	20150291954
RXR agonist	Methods and compositions for the treatment of cancer	Inhibition	β-catenin	Chemical	Allergan	20040009921
SIRT1 levels or activity	SIRTUIN BASED METHODS AND COMPOSITIONS FOR TREATING BETA-CATENIN-RELATED CONDITIONS	Inhibition	β-catenin deacetylation	Biomolecule	President and Fellows of Harvard College	20110009474
small molecule	CBP/CATENIN ANTAGONISTS FOR ENHANCING ASYMMETRIC DIVISION OF SOMATIC STEM CELLS	Inhibition	β-catenin-CBP	Chemical	University of Southern California	20150232468
small molecules	INHIBITORS OF BETA-CATENIN IN TREATMENT OF COLORECTAL CANCER	Inhibition	β-catenin-TCF4 complex	Chemical		20150374662
stapled alpha-helical peptides	Beta-catenin targeting peptides and uses thereof	Inhibition	β-catenin	Biomolecule	President and Fellows of Harvard College	8957026
TRPV4 inhibitor	PROTEIN INVOLVED IN DETECTION OF CANCER METASTASIS AND A TREATMENT THEREOF	Enhancer	β-catenin-E-cadherin	Chemical	National University of Singapore	20140199330
β-catenin peptides	Agents for treating human illnesses based on .beta.-catenin, and the production and use thereof	Inhibition	β-catenin-TCF complex	Biomolecule	Max-Delbruck-Centrum fur Molekulare Medizin	7067474
β-catenin protein	Methods for generating new hair follicles, treating baldness, and hair removal	Enhancer	β-catenin	Biomolecule	The Trustees of the University of Pennsylvania	9220926

Supplementary Table 1: USPTO Patent and Patent Applications
All others P/PA

Compound/Category	Title	Inhibitor or Enhancer	Molecular target	Interaction at level of:	Assignee	Patents and/or Patent Applications
Tankerase inhibitors	2-piperidin-1-yl-acetamide compounds for use as tankyrase inhibitors	Inhibition	Axin1/2 via tankerase	Axin1/2	Novartis	9181266, 9227982
Tankerase inhibitors	4-piperidinyl compounds for use as tankyrase inhibitors	Inhibition	Axin1/2 via tankerase	Axin1/2	Novartis AG	9163003
small molecule inhibitor	3-aryl-5-substituted-isoquinolin-1-one compounds and their therapeutic use	Inhibition	PARP1, TNKS1, TNKS2, etc	Axin1/2	Insititute of cancer research: Royal Cancer Hospital	9193689
small molecule inhibitor	Pyrvinium For The Treatment of Cancer	Inhibition	Pyrvinium	Axin1/2		20090099062
Azole derivatives	Azole derivatives as WTN pathway inhibitors	Inhibition	stabilize Axin2/destruction complex	Axin1/2	Oslo University Hospital HF	8883827, 9096587
nucleic acid, fushion protein, compound	METHODS AND COMPOSITIONS FOR MEASURING WNT ACTIVATION AND FOR TREATING WNT-RELATED CANCERS	Inhibition	Tankyrase-Axin	Axin1/2	Novartis	20100267626
Casein kinase inhibitors	BIFLUORODIOXALANE-AMINO-BENZIMIDAZOLE KINASE INHIBITORS FOR THE TREATMENT OF CANCER, AUTOIMMUNEINFLAMMATION AND CNS DISORDERS	Inhibition	Casein kinase 1 family	Casein Kinase 1	4SC DISCOVERY GMBH	20150158878
CKI polypeptides	Compositions and methods of diagnosis and treatment using casein kinase I	Inhibition	Wnt pathway	Casein Kinase 1	Chiron Corporation	6512102
DACT3, Wnt ligand inhibitors (Dkk-1, WIF-1, SFRPs)	Methods and compounds for preventing and treating a tumour	Inhibition	DACT3: Dvl2,	Dishevelled	Agency for Science, Technology and Research	8969313
6-bromo-indirubin (BIO), 5-amino-indirubin and N-methyl-indirubins and related indirubin derivatives	Indirubin-Type Compounds, Compositions, and Methods for Their Use	Enhancer	GSK3, CDKs, aryl hydorcarbon recpetors	GSK3		20070276025

Supplementary Table 2: Clincal Trials

Number	Identifier	Sponsor	Date Received	Condition	Intervention	target/method of action	Aliases	Status	Inhibition/Activation	Phase
1	NCT02278133	Array BioPharma	06-Oct-14	Metastatic CRC	Wnt974	Porcupine		recruiting	Inhibtion	Phase 1 Phase 2
2	NCT02649530	University of Michigan Cancer Center	05-Jan-16	Squamous Cell Carcinoma, Head And Neck	Wnt974	Porcupine		Not yet open	Inhibtion	Phase 2
3	NCT01351103	Novartis Pharmaceuticals	04-May-11	Pancreatic Adenocarcinoma; BRAF Mutant Colorectal Cancer; Other Tumor Types With Documented Genetic Alterations Upstream in the Wnt Signaling Pathway	LGK974	Porcupine		Suspended	Inhibtion	Phase 1
4	NCT02413853	University of Southern California	07-Apr-15	Metastatic CRC	PRI-724	CBP/β-catenin		not yet open	Inhibtion	Phase 2
5	NCT01323894	China Medical University Hospital	24-Mar-11	Primary Disease (Osteoporosis*)	Wnt3a	Activation of Wnt/β-catenin		Unknown	Activation	?
6	NCT02020291	WntResearch AB	11-Dec-13	Metastatic Breast Cancer; CRC; Prostate Cancer	Foxy-5	Frizzled receptor	Wnt5a mimic	completed	Inhibition of metastases	Phase 1
7	NCT01548066	Seoul National University Hospital	20-Jul-11	Androgenetic Alopecia; Male Pattern Baldness	Valproic Acid	GSK3b	Depakene	completed	Activation	Phase 2
8	NCT01764477	Prism Pharma Co., Ltd.	12-Dec-12	Pancreatic Adenocarcinoma	PRI-724	CBP/β-catenin		completed	Inhibition	Phase 1
9	NCT02655952	WntResearch AB	06-Jan-16	Metastatic: Breast, Colon, Prostate Cancer	Foxy-5	Frizzled receptor	Wnt5a mimic	not yet open	Inhibition of metastases	Phase 1b
10	NCT01398462	JW Pharmaceutical	17-Jul-11	Acute Myeloid Leukemia	CWP232291	β-catenin		completed	Inhibition	Phase 1
11	NCT02029352	Maastricht University Medical Center	18-Dec-13	Basal Cell Carcinoma	Topical Green Tea Ointment	Unspecified	epigallocatechin-3-gallate	completed	inhibition	Phase 2, Phase 3

Number	Identifier	Sponsor	Date Received	Condition	Intervention	target/method of action	Aliases	Status	Inhibition/Activation	Phase
12	NCT02687009	Michael Morse, MD	10-Feb-16	Colon Cancer	Niclosamide	Frizzled receptor inhibitor	Yomensan	not yet open	Inhibition	Phase 1
13	NCT02675946	Curegenix Inc	22-Jan-16	Advanced Solid Tumors (several)	CGX1321	Porcupine*		recruiting	Inhibition	Phase 1
14	NCT00088387	National Institute of Neurological Disorders and Stroke	23-Jul-04	Alzheimer's Disease	Lithium<br>Divalproex	GSK3b<br>GSK3b?	 Depakote	completed	activation	Phase 2
15	NCT02521844	D3 (Drug Product and Development), Biomedical Sciences Institute	27-Jul-15	Solid Tumors	ETC-1922159	Porcupine	ETC-159	recruiting	inhibition	Phase 1
16	NCT01606579	Prism Pharma Co., Ltd.	16-May-12	Acute/Chronic Myeloid Leukemia	PRI-724	CBP/β-catenin		Ongoing	Inhibition	Phase 1<br>Phase 2
17	NCT01214603	Eli Lily and Company	29-Sep-10	Leukemia	LY2090314	GSK3		completed	Activation	Phase 2
18	NCT01302405	Prism Pharma Co., Ltd.	18-Feb-11	Advanced Solid Tumors	PRI-724	CBP/β-catenin		terminated	inhibition	Phase 1
19	NCT01469975	Centre Leon Berard	03-Nov-11	Synovial Sarcoma	OTSA101-DTPA-90Y	Frizzled 10 neutralizing antibody	OTSA101 (unlabeled)	ongoing	Inhibition	Phase 1
20	NCT02503137	Samumed LLC	16-Jul-15	Androgenetic Alopecia	SM04554	Not specified		ongoing	Activation	Phase 2
21	NCT01985763	Randall F. Holcombe	09-Nov-13	CRC	Genistein	epigenetic modification of Sfrp2, Sfrp5and		Recruiting	Inhibition	Phase 1<br>Phase 2
22	NCT01457417	Leap Therapeutics	18-Oct-11	Multiple Myeloma; Solid Tumors; Non-Small Cell Lung Cancer	DKN-01	Dkk1 neutralizing antibody		completed	activation	Phase 1

Number	Identifier	Sponsor	Date Received	Condition	Intervention	target/method of action	Aliases	Status	Inhibition/Activation	Phase
23	NCT01608867	OncoMed Pharmaceuticals, Inc.	29-May-12	Solid Tumors	OMP-54F28	Wnt ligands		Ongoing	Inhibition	Phase 1
24	NCT01197209	Momotaro-Gene Inc.	03-Sep-10	Prostate Cancer	AD-REIC/Dkk3	LRP5/6	Dkk3	withdrawn	Inhibition	Phase 1
25	NCT01931046	Momotaro-Gene Inc.	21-Aug-13	Localized Prostrate Cancer	Ad5-SGE-REIC/Dkk-3	LRP5/6	Dkk3	recruiting		Phase 1 Phase 2
26	NCT02375880	Leap Therapeutics	18-Feb-15	Carcinoma of Intrahepatic and Extra-hepatic Biliary System; Carcinoma of Gallbladder; Bile Duct Cancer; Cholangiocarcinoma	DKN-01	Dkk1 neutralizing antibody		recruiting	activation	Phase 1
27	NCT00741377	Novartis Pharmaceuticals	22-Aug-08	Multiple Myeloma Bone Disease	BHQ880	Dkk1 neutralizing antibody		completed	activation	Phase 1
28	NCT01337752	Novartis Pharmaceuticals	12-Apr-11	Multiple Myeloma Renal Insufficiency	BHQ880	Dkk1 neutralizing antibody		completed	activation	Phase 2
29	NCT01302886	Novartis Pharmaceuticals	26-Jan-11	Smoldering Multiple Myeloma	BHQ880	Dkk1 neutralizing antibody		completed	activation	Phase 2
30	NCT01882660	Academisch Medisch Centrum - Universiteit van Amsterdam (AMC-UvA)	24-May-13	Colon Cancer	Decitabine	Wnt target genes derepression by demethylation		recruiting	activation	Not Applicable
31	NCT01293487	Pfizer	09-Feb-11	Osteopenia; Osteoporosis; Bone Disease	RN564	Dkk1 neutralizing antibody		completed	activation	Phase 1
32	NCT02013154	Leap Therapeutics	11-Dec-13	Esophageal Neoplasms; Adenocarcinoma of the Gastroesophageal Junction;. Gastroesophageal Cancer; Squamous Cell Carcinoma	DKN-01	Dkk1 neutralizing antibody		recruiting	activation	Phase 1

Number	Identifier	Sponsor	Date Received	Condition	Intervention	target/method of action	Aliases	Status	Inhibition/Activation	Phase
33	NCT01711671	Leap Therapeutics	18-Oct-12	Multiple Myeloma	DKN-01	Dkk1 neutralizing antibody		completed	activation	Phase 1 Phase 2
34	NCT01457417	Leap Therapeutics	18-Oct-11	Multiple Myeloma	DKN-01	Dkk1 neutralizing antibody		completed	activation	Phase 1
